# Dietary Supplementation with D-Ribose-L-Cysteine Prevents Hepatic Stress and Pro-Inflammatory Responses in Male Wistar Rats Fed a High-Fructose High-Fat Diet

**DOI:** 10.3390/pathophysiology29040049

**Published:** 2022-10-31

**Authors:** Abodunrin Adebayo Ojetola, Jerome Ndudi Asiwe, Wale Johnson Adeyemi, Dare Joshua Ogundipe, Adesoji Adedipe Fasanmade

**Affiliations:** 1 Department of Physiology, Faculty of Basic Medical Sciences, University of Ibadan, Ibadan 200005, Nigeria; 2 Department of Physiology, Faculty of Basic Medical Sciences, Redeemer’s University, Ede P.M.B 230, Nigeria; 3 Department of Physiology, Faculty of Basic Medical Sciences, Pamo University of Medical Sciences, Port Harcourt 500211, Nigeria; 4 Department of Physiology, Faculty of Basic Medical Sciences, Adeleke University, Ede 232104, Nigeria

**Keywords:** D-ribose-L-cysteine, oxidative stress, inflammation, liver, high-fat high-fructose

## Abstract

Diets rich in fats and fructose are associated with the pathogenesis of oxidative stress-induced non-alcoholic fatty liver disease. Therefore, we investigated the effect of D-ribose-L-cysteine (DRLC) in high-fructose high-fat (HFHF) diet-fed rats. Twenty rats (*n* = 5), divided into four groups, were simultaneously exposed to HFHF and/or DRLC (250 mg/kg) orally during the 8 weeks of the study. Results showed that HFHF precipitated pro-inflammation and selective disruption of the oxidative stress markers. There were significant decreases in the level of antioxidants such as superoxide dismutase (SOD), glutathione peroxidase (GPX), total antioxidant capacity (TAC), hepatic SOD and GPX. Significant increases in serum levels of uric acid (UA), tumour necrosis factor-alpha (TNF-α), C-reactive protein (CRP) and hepatic Xanthine oxidase (XO) were observed in the HFHF compared to the control. In the HFHF + DRLC group, oxidative stress was mitigated due to differences in serum levels of SOD, GPX, TAC, TNF-α, liver SOD, and XO relative to control. The administration of DRLC alone caused significant reductions in malondialdehyde, UA and CRP and a significant increase in SOD compared to the control. DRLC prevents hepatic and systemic oxidative stress and pro-inflammatory events in HFHF diet-fed rats.

## 1. Introduction

Diets rich in fructose and fats have been implicated in the pathogenesis of several diseases. Obesity, diabetes mellitus, hypertension, and non-alcoholic fatty liver diseases (NAFLD), to mention a few, have been reported as the direct consequences of excess intake of high-fructose and high-fat diets [1,2,3,4,5]. NAFLD incidence from dietary insult is associated with steatohepatitis and can progress to liver cirrhosis and hepatocellular carcinoma [4].

The aetiology of NAFLD and its related comorbidities have been linked to oxidative stress [6,7,8]. Oxidative stress is implicated in obesity, metabolic disorders, inflammatory diseases and cancer [9,10,11]. Despite the involvement of oxidative stress in the pathogenesis of metabolic disruptions that can initiate hepatic disorders, there is a dearth of reports on the use of antioxidants as an ameliorative or preventive regimen in this condition.

Previous reports have unveiled the potential of cysteine delivery agents such as homocysteine, methionine, N-acetylcysteine and L-cysteine in boosting GSH production [12,13,14] and enhancing the efficiency of the antioxidant system. However, there have been challenges associated with the delivery of the molecules in the biological system [15,16]. Diet-induced metabolic disruptions could be mitigated by cysteine delivery agents such as D-ribose-L-cysteine (DRLC). This dietary supplement enhances the endogenous synthesis of glutathione (GSH) [16,17,18,19], considered the most crucial molecule in the body responsible for detoxifying reactive radicals [20,21]. GSH is ubiquitously produced by all cells, but the liver is the primary source of GSH in humans [22]. Although it is utilised by most cells, the intestines, lungs and kidneys are the primary organs that use most of the GSH derived from the liver. It performs the role of proliferation and activation of cells, including lymphocytes and cytokines productions, thereby providing an adequate immune response in deficits [9].

Several studies have also examined other antioxidants’ physiological and biochemical roles, such as vitamins C and E, polyphenols, carotenoids, selenium, zinc and omega-3-fatty acids in different experimental animal models of obesity and metabolic syndrome [23,24,25,26,27]. Nevertheless, few studies have explored the therapeutic benefits of DRLC in diet-induced models of metabolic diseases. The benefits of L-cysteine on brain antioxidant levels in rats exposed to sodium valproate were previously documented [28]. It was also reported that DRLC mediates oxidative stress, spermatogenesis and steroidogenesis in diabetic rats [29]. The antidiabetic action of DRLC is comparable to that of insulin and selected oral hypoglycaemic agents in pregnant rats [30]. In addition, we reported the beneficial effects of DRLC in cardiometabolic syndrome following HFHF dietary exposure in rats [5].

However, in the present study, we investigated the long-term administration of DRLC and HFHF on hepatic function and inflammatory markers in male Wistar rats.

## 2. Materials and Methods

### 2.1. Drugs and Chemicals

Fructose was purchased from Qualikems Fine Chem Pvt. Ltd. Vadodara, Gujarat, India, while DRLC was obtained from Max International, Salt Lake City, UT, USA.

Diagnostic kits for the determination of thiol, superoxide dismutase (SOD), catalase (CAT), glutathione (GSH), glutathione peroxidase (GPX), total antioxidant capacity (TAC), malondialdehyde (MDA), uric acid (UA), tumour necrosis factor*–*alpha (TNF-α) and c-reactive protein (CRP) were purchased from Fortress Diagnostics Limited, Antrim, Northern Ireland, UK. However, analytical kits for the determination of albumin (ALB), globulin (Glob), gamma-glutamyltransferase (GGT) and xanthine oxidase (XO) were procured from Elabscience Biotechnology Company, Ltd., Wuhan, Hubei, China.

### 2.2. Animal Care

The twenty adult male Wistar rats (weight range: 200–220 g) used for this study were purchased from the Central Animal House of the University of Ibadan, Ibadan, Nigeria. The animals were kept in plastic cages with wood shaving under standard environmental conditions in the Animal House of the Faculty of Basic Medical Sciences, University of Ibadan. The rats were acclimatised for one week before random allotment to separate groups. Rodent pellets (manufactured by: Top feeds Ltd., Ibadan, Nigeria) and water were given ad libitum daily, and the animals were weighed weekly with the aid of a digital weighing scale (manufacturer: Zhejiang Haoyu Industry & Trade Co., Ltd. Zhejiang, China). Prior to the commencement of the study, ethical approval was obtained from the Animal Care and Use Research Ethics Committee of the University of Ibadan, Nigeria. It was also conducted following the “Guide for the Care and Use of Laboratory Animals” documented by the National Academy of Sciences [31].

### 2.3. Feed Formulation

A high-fructose, high-fat diet was formulated with lard (20%) and fructose (17%) added to 63% of standard rat chow as previously reported [5].

### 2.4. Experimental Design

The rats were randomly divided into four (4) groups, namely: Control (group 1); high-fructose high-fat (HFHF) (group 2); HFHF + DRLC (group 3) and DRLC (group 4). Group 1 was fed standard rat chow (SRC) and orally administered distilled water (1 mL/kg BW/day). Groups 2 and 3 were fed with an HFHF diet in addition to their daily exposure to 10% fructose in the drinking water; however, group 3 was also administered DRLC (250 mg/kg/day) orally [19] during the 8 weeks duration of the study. Group 4 was orally given SRC and DRLC (250 mg/kg/day).

### 2.5. Termination of the Experiment

Twelve hours after administration on the last day of the 8 weeks study, the rats were administered sodium pentobarbital (40 mg/kg, intraperitoneally., manufacturer: Nicholas Piramal Limited, London, UK) anaesthesia [32,33]. After that, they were dissected, and blood was collected by cardiac puncture. The blood in heparinised sample bottles was centrifuged at 1372× *g* for 15 min at 4 °C using a cold centrifuge (Centurion Scientific Ltd., West Sussex, United Kingdom). The resultant supernatant plasma samples were pipetted into separate plain bottles. The liver tissue of the rats was excised, and a uniform portion (100 mg) was homogenised in 10% *w/v* ice-cold 0.1 M phosphate buffer (pH 7.4). The resultant homogenate was used for the estimation of some biochemical parameters. The biochemical profile in the serum and the liver tissue was determined using diagnostic kits based on the manufacturer’s instructions.

### 2.6. Statistical Analysis

Data were analysed using Graphpad prism (version 7.0) for Windows (GraphPad Software, San Diego, CA, USA). Statistical evaluations of the differences between the group mean values were tested by one-way analysis of variance, followed by Tukey post hoc test for multiple comparisons. The results were expressed as mean ± standard error of mean (SEM), and statistical significance was considered at *p* ≤ 0.05.

## 3. Results

### 3.1. Effects of D-Ribose-L-Cysteine (DRLC) on Antioxidant/Pro-Oxidant Indices in the Serum of Male Wistar Rats Fed with High Fat-High Fructose (HFHF) Diet

Even though significant differences were not recorded in the activities of catalase (CAT), glutathione peroxidase (GPX) and glutathione (GSH), reductions in superoxide dismutase (SOD), GPX and total antioxidant capacity (TAC) were observed in group 2 relative to the control (*p* < 0.01, *p* < 0.0001 and *p* < 0.02, respectively). GSH value of the HFHF group was low compared to the control but not to a significant level (*p* < 0.07). (Table 1). Moreover, compared to the latter, there was a significant increase and decrease in SOD (*p* < 0.0001) and malondialdehyde (MDA) (*p* < 0.0003) levels, respectively, in group 4. Moreover, there was a significant reduction in MDA level in group 4, relative to group 2 (Table 1). In comparison to the HFHF group, it was noted that SOD and GPX activities were significantly increased in groups 3 and 4 (*p* < 0.0028, *p* < 0.0002, respectively), although the MDA level was significantly reduced. Furthermore, we observed a significant elevation and reduction in SOD and MDA levels, respectively, in group 4, relative to group 3 (*p* < 0.0001, *p* < 0.0004, respectively) (Table 1).

### 3.2. Effects of D-Ribose-L-Cysteine (DRLC) on Inflammatory Indices and Selected Enzymes in the Serum of Male Wistar Rats Fed with High Fat-High Fructose (HFHF) Diet

**Figure 1:**
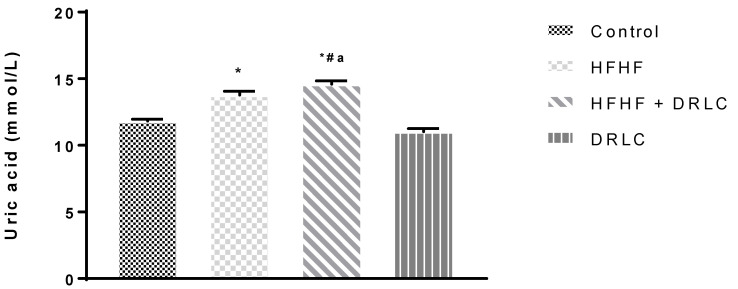
Serum Uric acid. *n* = 5. Values were expressed as Mean ± SEM, * significantly different from control (*p* < 0.05); **^#^** significantly different from HFHF (*p* < 0.05); ^a^ significantly different from HFHF + DRLC (*p* < 0.05). NB: Control, HFHF (high fructose high fat), HFHF + DRLC (D-ribose-L-cysteine) and DRLC in Wistar rats after 8 weeks of DRLC treatment.

**Figure 2:**
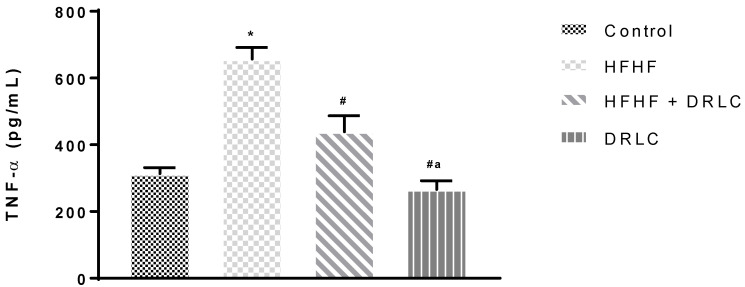
Serum TNF-α (Tumour necrosis factor—alpha). *n* = 5. Values were expressed as Mean ± SEM, * significantly different from control (*p* < 0.05); **^#^** significantly different from HFHF (*p* < 0.05); ^a^ significantly different from HFHF + DRLC (*p* < 0.05). NB: Control, HFHF (high fructose high fat), HFHF + DRLC (D-ribose-L-cysteine) and DRLC in Wistar rats after 8 weeks of DRLC treatment.

**Figure 3:**
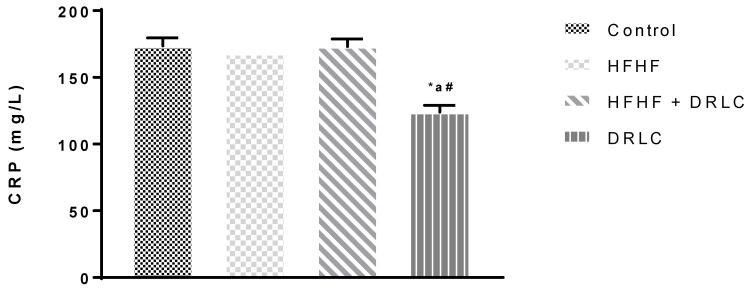
Serum CRP (C-reactive protein). *n* = 5. Values were expressed as Mean ± SEM, * significantly different from control (*p* < 0.05); **^#^** significantly different from HFHF (*p* < 0.05); ^a^ significantly different from HFHF + DRLC (*p* < 0.05). NB: Control, HFHF (high fructose high fat), HFHF + DRLC (D-ribose-L-cysteine) and DRLC in Wistar rats after 8 weeks of DRLC treatment.

Across the groups, there was no significant difference in albumin (ALB), globulin (GLOB), alanine aminotransferase (ALT) and alkaline phosphatase (ALP) levels (Table 2). Nevertheless, the uric acid (UA) (Figure 1) and tumour necrotic factor-alpha (TNF-α) (Figure 2) levels were significantly increased in group 2 when compared to the control group (*p* < −0.0001 and *p* < 0.0001, respectively). TNF-α of group 3 was not significantly different from the control (*p* < 0.07). It should be noted that, relative to the control group, we recorded a significant increase in aspartate aminotransferase (AST) activity (Table 2) in group 3 (*p* < 0.02) and a significant reduction in UA in group 4 (*p* < 0.0001) (Figure 1). Compared to the HFHF group, there was a significant reduction in TNF-α in group 3 (*p* < 0.0016). In addition, relative to the HFHF group, significant reductions in UA and TNF-α were documented in group 4 (*p* < 0.0001, *p* < 0.0001, respectively). Compared to the latter, significant elevations in UA and TNF-α were noted in group 3 (*p* < 0.0001 and *p* < 0.0001, respectively). There was a significant reduction in the CRP level of group 4 compared to the control, HFHF and HFHF + DRLC groups (*p* < 0.0001, *p* < 0.0002, *p* < 0.0001, respectively) (Figure 3).

### 3.3. Effects of D-Ribose-L-Cysteine (DRLC) on Hepatic Oxidative Indices, Absolute and Relative Liver Weight in Male Wistar Rats Fed with High Fat-High Fructose (HFHF) Diet

In the HFHF group, there were significant reductions in hepatic SOD and GPX and significant increases in liver weight and xanthine oxidase (XO), relative to the control group (*p* < 0.05, *p* < 0.03, *p* < 0.0001 and *p* < 0.05, respectively) (Table 3). Moreover, for the latter, a significant reduction and increase in GPX activity and relative liver weight were recorded in group 3 (*p* < 0.01 and *p* < 0.03, respectively). In comparison to the HFHF group, there was a significant increase in glutathione (GSH), but significant reductions in liver gamma-glutamyl transferase (GGT), XO and weight in group 4 (*p* < 0.05,, *p* < 0.05, *p* < 0.01 and *p* < 0.02 respectively) (Table 3). The GPX activity in group 4 was significantly increased compared to group 3 (*p* < 0.03).

## 4. Discussion

Studies have shown the association between oxidative stress and diets rich in fats and fructose [34,35]. DRLC is a pro-drug that enhances the synthesis of GSH. This master antioxidant plays a prominent role in regulating the activities of cellular antioxidants and providing protection against oxidative stress. A previous study reported that DRLC caused a significant increase in the level of GSH in different brain regions of mice exposed to lipopolysaccharide [36]. In agreement with this report that links DRLC and GSH, this study also observed that prolonged administration of DRLC in animals fed with HFHF diet resulted in elevated hepatocytes GSH. Unlike observed changes in the hepatic level of GSH across the animal groups, there was no significant difference in the serum level of the biomarker across the animal groups. This may be partly due to the primary responsibility of hepatic tissue in GSH synthesis and reports indicating its predominance, particularly in liver cells [22,37,38]. Moreover, stringent feedback mechanisms involved in the regulations of GSH at the systemic level might also be responsible for keeping GSH levels within a physiological range.

Exposure to HFHF caused oxidative stress, evidenced by selective reductions in the activities of SOD and GPX in the hepatic tissue but not CAT and GSH. The increase in SOD of the group administered DRLC alone may be associated with the administration of DRLC. An increase in SOD activities is an essential physiological indicator reflecting protection afforded to the body to prevent inflammatory responses and oxidative stress [39,40]. Hepatic SOD increases in the treated group agree with a previous study that reported an increase in heart GSH and SOD in Wistar rats fed concurrently with HFHF and DRLC [5]. In addition, SOD activity was similarly increased due to DRLC administration in pregnant diabetic rats [30]. This indicates that DRLC potentiates the ability to increase antioxidant enzymes such as SOD in liver and heart tissues, especially in oxidative stress-induced disorders. Additionally, these variations were consistent with the observed level of these biomarkers in the plasma in this study. The observed compromised integrity of the antioxidant system following HFHF dietary exposure was further affirmed by the significant reduction in serum TAC of the HFHF group relative to the control. This further showed that DRLC supplementation in a homeostatic system complements the endogenous antioxidant system in getting rid of reactive species produced secondary to various normal metabolic activities in the biological system. The significant differences in the plasma SOD, MDA, and liver XO in the DRLC group relative to the control and HFHF groups provide evidence of the anti-oxidative action of DRLC. It is noteworthy that this effect seems to be peculiar at the systemic level, not the hepatic level. Although CAT, GSH and MDA activities were unchanged in the HFHF group, the increase in hepatic XO reflects the pro-oxidative event, which was abated through DRLC administration. So, these are indications that the mechanism through which DRLC restored the integrity of the antioxidant system might be associated with its cysteine-derived GSH biosynthesis [41].

Assessments of the level of activities of ALT, AST, ALP and GGT have been used to evaluate the integrity of the hepatic tissue [33], as damaged hepatic cells are known to release transaminases into the systemic circulation. The insignificant differences in the activities of ALT, AST and ALP in the HFHF group relative to the control group showed functional preservation of hepatic tissue following HFHF exposure. This was further illustrated by the insignificant differences in the plasma GLOB and ALB when comparisons were made between the aforementioned groups, an attestation of uncompromised synthesis of plasma protein synthesised in the hepatic tissue.

The antioxidant effect of DRLC partially explains its inflammatory action, as these two endogenous processes are related. With favourable effects on indices of oxidative stress through SOD, MDA and XO in the DRLC group, compared to the control and HFHF groups, the dietary supplement abated pro-inflammatory events that accompany metabolic pro-oxidative processes in rats fed with standard rat chow and those exposed to HFHF. This was demonstrated by the significant decreases in the level of UA and TNF-α in the DRLC group relative to HFHF and control groups. From our previous study, DRLC prevented an exponential increase in body weight after exposure to a HFHF diet [5]. This attribute could arguably be linked to its ability to prevent the accumulation of uric acid. Thus, its ability to improve antioxidant capacity and prevent pro-inflammatory conditions might be associated with the prevention of hyperuricaemia because fructose inclusion in diet triggers hyperuricaemia. In the present study, the significant increase in the level of UA in the HFHF group relative to the control group was complemented by a significant elevation in XO in the former compared to the latter. A positive association between UA and XO has been reported. The latter is known to increase the generation of ROS by converting hypoxanthine to xanthine, during which UA is produced [42]. The development of metabolic disorders following high fructose/fat exposure is characterised by hyperuricaemia [43,44]. This pro-inflammatory effect of HFHF exposure was justified by the significant elevations in the plasma levels of UA and TNF-α, but not CRP in the HFHF group relative to the control group. A population-based survey revealed a strong correlation between serum UA and CRP levels, [45] and experimental studies opined that UA stimulates pro-inflammatory responses and up-regulates the synthesis and release of inflammatory markers such as interleukin-6 and TNF-α [46]. This study further demonstrated the anti-inflammatory effect of DRLC previously reported [34] by the significant reduction in UA and TNF-α in the HFHF + DRLC group to levels comparable to the control group.

Moreover, DRLC significantly decreased TNF-α in the HFHF + DRLC group relative to the HFHF. This might also suggest that the concurrent administration of DRLC with the HFHF diet supports a response to decrease inflammation that may be generated through a HFHF diet. Reduced circulating CRP could indicate decreased CRP production by the hepatocytes, suggesting decreased or absence of inflammation by other body tissues. A decrease in CRP level following administration of DRLC only might also suggest its ability to inhibit inflammatory responses in Wistar rats treated with DRLC, which might be dose-response related.

The present study showed that chronic dietary exposure to HFHF resulted in pro-inflammation, pro-oxidative events and NAFLD. Although there was hepatic oxidative stress and pro-inflammatory activities, there were no alterations in liver protein and enzymes. In the homeostatic state, DRLC neither disrupted the synthesis of hepatic protein nor affected the activities of hepatic enzymes. However, it showed the potential to prevent oxidative stress and pro-inflammatory events. Thus, through DRLC, there was significant mitigation of HFHF-diet-induced pathophysiological processes.

## 5. Conclusions

D-ribose-L-cysteine increased selective hepatic and systemic antioxidant capacities through GSH supplementation, thereby preventing oxidative stress in Wistar rats fed a high-fructose high-fat diet. Additionally, DRLC reduced the CRP level in male Wistar rats.

## Figures and Tables

**Table 1:** Effects of D-ribose-L-cysteine (DRLC) on antioxidant/pro-oxidant indices in male Wistar rats fed with high fructose-high fat (HFHF) diet.

Groups/Parameters	SOD (u/mL)	CAT (μmol/min/mL)	GSH (mM)	GPX (u/L)	TAC (mmol/mL)	MDA (μM)
1. Control	0.16 ± 0.01	17.97 ± 0.72	1.24 ± 0.14	1.90 ± 0.16	1.75 ± 0.17	3.39 ± 0.32
2. HFHF	0.04 ± 0.02 *	16.42 ± 0.84	0.77 ± 0.26	0.35 ± 0.04 *	0.79 ± 0.14 *	3.35 ± 0.43
3. HFHF + DRLC	0.19 ± 0.03 ^#^	18.29 ± 0.37	0.97 ± 0.19	1.42 ± 0.15 ^#^	1.33 ± 0.32	3.33 ± 0.38
4. DRLC	0.39 ± 0.03 *^#a^	18.81 ± 0.72	1.49 ± 0.21	1.61 ± 0.14 ^#^	1.44 ± 0.14	0.83 ± 0.12 *^#a^

Values were expressed as mean ± SEM. * *p *< 0.05 is significant compared to control group; ^#^ *p *< 0.05 is significant compared to HFHF group; ^a^ *p *< 0.05 is significant—DRLC + HFHF vs. DRLC.NB: SOD—Superoxide dismutase; CAT—Catalase; GSH—Glutathione; GPX—Glutathione peroxidase; TAC—Total antioxidant capacity; MDA—Malondialdehyde.

**Table 2:** Effects of D-ribose-L-cysteine (DRLC) on hepatic enzymes and proteins in male Wistar rats fed with high fructose-high fat (HFHF) diet.

Groups/Parameters	ALB (g/dL)	GLOB (g/dL)	ALT (mg/dL)	ALP (mg/dL)	AST (mg/dL)
1. Control	2.92 ± 0.09	4.18 ± 0.06	14.44 ± 0.41	48.46 ± 0.54	4.72 ± 0.21
2. HFHF	3.48 ± 0.15	4.24 ± 0.12	15.69 ± 0.59	47.13 ± 0.06	5.62 ± 0.23
3. HFHF + DRLC	3.32 ± 0.19	4.08 ± 0.04	15.07 ± 0.66	48.73 ± 0.68	6.08 ± 0.28 *
4. DRLC	3.22 ± 0.22	4.08 ± 0.12	15.26 ± 0.19	46.66 ± 0.60	5.22 ± 0.37

Values were expressed as mean ± SEM. * *p *< 0.05 is significant compared to control group. NB: UA—Uric acid; TNF-α—Tumour necrotic factor—alpha; CRP—c-reactive protein; ALB—Albumin; GLOB—Globulin; ALT—Alanine aminotransferase; ALP—Alkaline phosphatase; AST—Aspartate aminotransferase.

**Table 3:** Effects of D-ribose-L-cysteine (DRLC) on hepatic tissue oxidative indices, absolute and relative liver weight in male Wistar rats fed with high fructose-high fat (HFHF) diet.

Groups/ Parameters	Liver SOD (U/mL)	Liver CAT (μmol/min/mL)	Liver GSH (mM)	Liver GPX (U/L)	Liver THIOL (nmol/mg)	Liver GGT (U/L)	Liver XO (U/L)	Liver Weight (g)	Relative Liver Weight (%)
1. Control	0.78 ± 0.07	6.72 ± 0.52	3.92 ± 0.47	4.30 ± 0.57	3.58 ± 0.59	4.45 ± 0.81	18.74 ± 1.79	6.47 ± 0.30	2.41 ± 0.15
2. HFHF	0.42 ± 0.05 *	6.73 ± 0.65	2.33 ± 0.52	1.63 ± 0.30 *	3.09 ± 0.64	4.95 ± 0.98	28.55 ± 2.49 *	8.29 ± 0.12^*^	2.69 ± 0.07
3. HFHF + DRLC	0.53 ± 0.14	4.43 ± 0.92	2.56 ± 0.33	0.98 ± 0.22 *	3.68 ± 0.29	2.60 ± 0.45	22.29 ± 3.68	7.52 ± 0.43	2.96 ± 0.15 *
4. DRLC	0.55 ± 0.07	5.95 ± 0.64	4.11 ± 0.54 ^#^	3.52 ± 0.95 ^a^	4.15 ± 0.85	2.35 ± 0.57^#^	15.14 ± 2.47 ^#^	6.95 ± 0.14 ^#^	2.67 ± 0.11

Values were expressed as mean ± SEM. * *p *< 0.05 is significant compared to control group; ^#^ *p *< 0.05 is significant compared to HFHF group; ^a^ *p *< 0.05 is significant—DRLC + HFHF vs. DRLC. NB: SOD—Superoxide dismutase; CAT—Catalase; GSH—Glutathione; GPX—Glutathione peroxidase; GGT—Gamma-glutamyltransferase; XO—Xanthine oxidase.

## Data Availability

The datasets used and/or analysed during the current study are available from the corresponding author on reasonable request.
